# Consumption of Milk-Protein Combined with Green Tea Modulates Diet-Induced Thermogenesis

**DOI:** 10.3390/nu3080725

**Published:** 2011-07-27

**Authors:** Rick Hursel, Margriet S. Westerterp-Plantenga

**Affiliations:** Department of Human Biology, Nutrition and Toxicology Research Institute Maastricht (NUTRIM), Maastricht University, P.O. Box 616, Maastricht 6200 MD, The Netherlands; Email: m.westerterp@maastrichtuniversity.nl

**Keywords:** obesity, catechins, polyphenol-protein complexes, energy expenditure

## Abstract

Green tea and protein separately are able to increase diet-induced thermogenesis. Although their effects on long-term weight-maintenance were present separately, they were not additive. Therefore, the effect of milk-protein (MP) in combination with green tea on diet-induced thermogenesis (DIT) was examined in 18 subjects (aged 18–60 years; BMI: 23.0 ± 2.1 kg/m^2^). They participated in an experiment with a randomized, 6 arms, crossover design, where energy expenditure and respiratory quotient (RQ) were measured. Green tea (GT)*vs.* placebo (PL) capsules were either given in combination with water or with breakfasts containing milk protein in two different dosages: 15 g (15 MP) (energy% P/C/F: 15/47/38; 1.7 MJ/500 mL), and 3.5 g (3.5 MP) (energy% P/C/F: 41/59/0; 146.4 kJ/100 mL). After measuring resting energy expenditure (REE) for 30 min, diet-induced energy expenditure was measured for another 3.5 h after the intervention. There was an overall significant difference observed between conditions (*p* < 0.001). Post-hoc, areas under the curve (AUCs) for diet-induced energy expenditure were significantly different (*P* ≤ 0.001) for GT + water (41.11 [91.72] kJ・3.5 h) * vs.* PL + water (10.86 [28.13] kJ・3.5 h), GT + 3.5 MP (10.14 [54.59] kJ・3.5 h) and PL + 3.5 MP (12.03 [34.09] kJ・3.5 h), but not between GT + 3.5 MP, PL + 3.5 MP and PL + water, indicating that MP inhibited DIT following GT. DIT after GT + 15 MP (167.69 [141.56] kJ・3.5 h) and PL + 15 MP (168.99 [186.56] kJ・3.5 h) was significantly increased *vs.* PL + water (*P* < 0.001), but these were not different from each other indicating that 15 g MP stimulated DIT, but inhibited the GT effect on DIT. No significant differences in RQ were seen between conditions for baseline and post-treatment. In conclusion, consumption of milk-protein inhibits the effect of green tea on DIT.

## 1. Introduction

The growing prevalence of obesity worldwide is becoming one of the main factors contributing to increased mortality. As people consume more energy than they can expend, weight gain is the consequence [1]. Natural and healthy ingredients, such as green tea and proteins, can prevent weight gain or even induce weight loss when consumed in adequate amounts. Green tea has been shown to increase 24 h and long-term energy expenditure and fat oxidation and also has a positive effect on body-weight regulation [2,3,4,5]. Proteins as well as green tea have the ability to enhance energy expenditure, but it is still unknown whether administration of catechins simultaneously with a meal that contains protein will enhance energy expenditure more than protein by itself. Recently, we showed that when given together, green tea and protein have no synergistic effect. After a 4 week intervention with a very low energy diet followed by a 3 month weight-maintenance period, similar results were found in subjects on a high-protein diet plus green tea-caffeine mixture as in subjects receiving either a high-protein diet plus placebo or an adequate protein diet plus green tea-caffeine mixture [6]. One possible explanation for the observed lack of synergistic effect is due to the formation of complexes between proteins and the polyphenols in tea, which was first reported in 1963 by Brown and Wright [7]. In particular caseins, which are present in milk protein (MP), tend to bind the polyphenols. Several studies have confirmed the formation of such complexes where the protein “wraps” itself around the catechins, a process named non-covalent cross-linking [8,9,10,11]. This process might reduce the bioavailability and accessibility of the polyphenols [12]. Several studies examined the difference in anti-oxidative and anti-mutagenic capacity between tea with or without the addition of milk [13,14,15,16,17,18,19,20,21]. Serafini *et al.* [13] found that total anti-oxidant capacity did not decrease due to the addition of milk to tea, but the polyphenols were rather unavailable for absorption as the polyphenol-protein complexes were resistant to gastric hydrolysis. Other studies did not find a difference in the anti-oxidant capacity of tea after the addition of milk compared to tea alone [18,20,21,22].

Hence, the aim of this study was to examine the effect on diet-induced thermogenesis of a combination of different amounts of milk-protein and green tea.

## 2. Subjects and Methods

### 2.1. Subjects

Eighteen subjects (9 females, 9 males) participated in this study, they were healthy, aged 18 to 60 years and had a BMI between 20 and 33 kg/m^2^. The subjects were recruited by advertisements in local newspapers and on notice boards at the university. All volunteers (*N* = 94) participated in an initial screening that involved measurements of body weight and height and included the completion of a questionnaire related to eating behavior (Three Factor Eating Questionnaire, TFEQ [23]) and the completion of a questionnaire related to health, use of medication, physical activity, alcohol consumption, food allergies, smoking behavior and daily caffeine consumption. All subjects were in good health, non-smokers, not using medication (except for contraception), at most moderate alcohol consumers and unrestrained eaters (as assessed by factor 1 of the TFEQ). All subjects gave their written informed consent and the Medical Ethical Committee of Maastricht University approved the study.

### 2.2. Experimental Design

The study had a randomized, six arms, single-blind, crossover design. Subjects attended the university-laboratory once a week, for six consecutive weeks. They were instructed to abstain from caffeine-rich products like tea, coffee, cola-type soft drinks and energy drinks. They traveled by public transport or car, in order to avoid physical activity that would have increased resting energy expenditure (REE). Subjects arrived in the fasted state at 08:15 h and were kept in a time-blinded surrounding. They emptied their bladder before the test. After resting on a bed for 30 min, the REE and the substrate oxidation of the subjects was measured for 30 min by means of an open-circuit, ventilated-hood system. Gas analysis was performed by a paramagnetic oxygen analyzer (omnical type 1155B, Crowborough Sussex, UK) and an infrared carbon dioxide analyzer (omnical type 1520/1507). Energy expenditure was calculated using Weir’s formula [24]. The RQ was calculated as CO_2_ produced/O_2_ consumed. The subjects were lying in the supine position. 

This study tested the effect of GT *vs.* PL on diet-induced thermogenesis with or without different amounts of milk-protein. Subjects received in random order one of the six interventions after measuring the REE, consisting of either water (100 mL), 3.5 g MP or 15 g MP, in addition to which the subjects ingested three GT capsules (Sunphenon^®^ 90 LB, Taiyo Kagaku Co. Ltd, Mie, Japan), or the control, which were three PL capsules (Gelkaps, Falkenhagen, Germany). The 3.5 g of MP were obtained from a 100 mL dairy product (FrieslandCampina, Leeuwarden energy% P/C/F: 41/59/0; 146.4 kJ/100 mL). The 15 g MP were obtained from a dairy product (FrieslandCampina, Leeuwarden, energy% P/C/F: 15/47/38). Before the study started, ratings for palatability and acceptability were assessed with visual analogue scales. With an average rating of 68 mm, subjects rated the palatability as sufficient. Hedonics was not different between drinks. The 15 MP dairy products were subject-specifically calculated, by calculating basal metabolic rate (BMR) of each subject with the Harris and Benedict equation, which uses gender, age, height and weight [25]. To estimate the total energy need, the outcome of the equation was multiplied with a physical activity index [26] of 1.6, estimated by means of a computer simulation program [27]. The 15 MP dairy products contained 15% of the daily energy need and had an energy density of 3.2 kJ/g. Energy content varied from 1.4 to 2.3 MJ, with an average of 1.7 MJ. The capsules all had the same appearance. The composition and the dose of the capsules are presented in Table 1. During the consumption of the intervention the hood was removed temporarily. After the intervention the hood was placed back and the measurements continued for another 3.5 h, during which the diet-induced thermogenesis was determined. Subjects were not allowed to talk, laugh, move or sleep while lying under the hood [28]. 

**Table 1:** Composition of the green tea capsules (mg).

	GT	PL
**Total polyphenols**	207.1	
**Total catechins**	169.0	
**Epigallocatechin gallate**	84.5	
**Caffeine**	2.1	
**Soy oil**	316.9	528.2
		
**Total filling weight (mg/capsule)**	528.2	528.2
**Total weight (mg/capsule)**	757.0	757.0
**Total weight (mg/testday)**	1584.6	1584.6

PL, placebo; GT, green tea; GT: Sunphenon 90 LB (Taiyo Kagaku Co. Ltd., Mie, Japan) decaffeinated green tea extract.

### 2.3. Statistical Analysis

Data are presented as median plus range, unless otherwise indicated. Diet-induced thermogenesis was calculated as iAUC (area above baseline), by the conventional trapezoid method. The baseline measurement (REE) was subtracted from the total area under the curve. Data were analyzed using PASWStatistics 18.0 (SPSS Inc. Chicago, Illinios, USA). Initially, the data were tested for normality with the Kolmogorov-Smirnov test. Data were skewed, therefore differences were analyzed non-parametrically. Non-parametric tests such as Mann-Whitney U (post-hoc analysis) and Kruskall-Wallis (overall significance) were used to determine a possible difference between effects of the six interventions on diet-induced thermogenesis. *P* values obtained after Mann-Whitney U tests underwent Bonferroni correction. Factor DIT, which expresses DIT after correction for body size, was calculated as follows: (REE + DIT)/REE [29]. The level for establishing significant differences was taken at *p* < 0.05, after Bonferroni correction the adjusted *P*-value for significance was *p* < 0.003.

## 3. Results

No adverse events occurred as no subjects reported any feelings of discomfort after consuming the interventions and the capsules. No different effects for men or women were observed; therefore these data have been taken together. 

**Figure 1:**
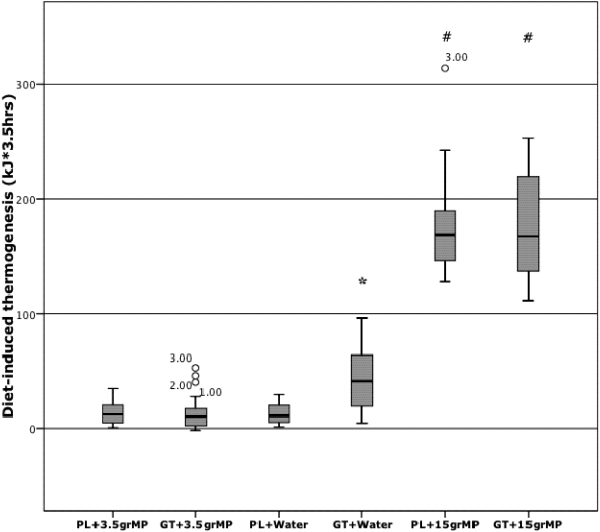
Areas under the curve (AUCs) for energy expenditure after the GT + water, PL + water, GT + 3.5 MP, PL + 3.5 MP, GT + 15 MP and PL + 15 MP condition in 18 subjects (men and women). Data are presented as box-and-whisker plots and values are expressed as medians plus range (minimal, maximal). Mann-Whitney U test. * *P*≤0.001, # *P* < 0.001.

A significant overall treatment (*p* < 0.001) effect for energy expenditure after the interventions was obtained. Areas under the curve for energy expenditure were significantly different for GT + 15 MP (167.69 [141.56] kJ・3.5 h) and PL + 15 MP (168.99 [186.56] kJ・3.5 h) compared with GT + water (41.11 [91.72] kJ・3.5 h), PL + water (10.86 [28.13] kJ・3.5 h), GT + 3.5 MP (10.14 [54.59] kJ・3.5 h) and PL + 3.5 MP (12.03 [34.09] kJ・3.5 h) (P < 0.001). Furthermore, GT + water differed significantly from PL + water, GT + 3.5 MP and PL + 3.5 MP (P ≤ 0.001). No significant differences were found between GT + 15 MP and PL + 15 MP and also not between PL + water, GT + 3.5 MP and PL + 3.5 MP as presented in box-and-whisker plots (Figure 1). Post-hoc, DIT after GT + water was significantly increased compared to DIT after PL + water (*P* < 0.001), indicating that GT alone stimulated DIT. However, DIT after PL + 3.5 MP was not increased compared to PL + water (*P* = 0.09), nor was DIT after GT + 3.5 MP (*P* = 0.09), indicating that 3.5 MP inhibited the GT alone effect on DIT. Furthermore, both DIT after PL + 15 MP as well as DIT after GT + 15 MP were significantly increased , compared to PL + water (*P* < 0.001), indicating that 15 MP stimulated DIT. However, GT + 15 MP was not different from PL + 15 MP (*P* = 0.66), indicating that MP prevented an additional GT effect on DIT. 

No gender-differences were seen after comparing the percentages of all conditions between males and females. This was also shown after correction for body size by means of calculating factor DIT, where outcomes for energy expenditure remained similar between the interventions. 

Furthermore, no significant differences between conditions were found for RQ and macronutrient balances.

## 4. Discussion

After examining the effect of a combination of green tea with different amounts of dietary protein, the results show that no additional effect occurs when green tea and milk protein are simultaneously administered. GT + 15 MP had a similar significant effect on DIT compared to PL + 15 MP. Yet, since the DIT of GT + water was significantly elevated, we may conclude that 15 MP inhibited a possible additional effect of GT. Furthermore, GT + water increased DIT compared with PL + water, GT + 3.5 MP or PL + 3.5 MP. Apparently there is no synergistic effect from catechins and milk protein when given simultaneously, regardless of the amount. The diet-induced thermogenesis from milk protein, for 3.5 MP and 15 MP alone, was not elevated by the catechins to a higher extent, as compared to placebo (GT + water *vs.* PL + water) [2]. With GT + 15 MP the milk protein prevented an additional effect, thereby this condition showed a similar effect compared with PL + 15 MP. Even small amounts of milk protein (3.5 g) were sufficient to suppress the effect of green tea on diet-induced thermogenesis. Milk protein reduced the average post-meal AUC over 3.5 h from 42 kJ to 14.5 kJ, without an appreciable increase in DIT attributable to the milk itself. These results were still present when corrected for multiple testing. With respect to fat oxidation, previous studies did find an effect of green tea on fat oxidation [2]. However, there was no additive effect of green tea on fat oxidation seen in the current study, since the differences between baseline and post-treatment RQ values did not differ. It could be that with GT, mainly dietary fat oxidation is stimulated, and that this effect was not shown in the present study because the breakfast contained hardly any or very little fat.

Previously, in concurrence with the theory of Brown and Wright [7] we reported that the formation of complexes between catechins and proteins, in particular the proline-rich β-casein that is preferred by polyphenols, prevented a synergistic effect of catechins and green tea after 3 months of weight maintenance. Controversy remains about whether combining proteins with tea inhibits the beneficial effect of tea drinking, and different mechanistic explanations exist for this phenomenon [13,14,15,16,17,18,19,20,21]. For instance, the addition of protein increases the pH of the stomach resulting in a reduced absorption of catechins and affects the weak acid compounds of the polyphenols. Polyphenols are easily absorbed in their non-ionized form, but a rise in stomach pH increases the ionization thereby impeding the passage of the polyphenols through the gastric mucosa [13]. This suggestion leads to another possible explanation for the lack of a synergistic effect in the current study, namely, that the change of pH in the stomach by the proteins would make it impossible for the casing of the capsules to dissolve. Nevertheless, this seems rather unlikely because the raw material of which the casing consisted was gelatin. This is a common material for producing coating/casing of capsules. 

Some other studies did not find an inhibition of the anti-oxidant capacity of tea after the addition of proteins. Therefore, Van het Hof *et al.* suggest that not the protein content itself is the cause of the inhibition but possibly the fat content. Catechins have a complex ring structure and are fat-soluble which causes the formation of complexes between fat and polyphenols [18]. This was contradicted by Krul *et al.* who examined three types of milks (whole milk, semi-skimmed milk and skimmed milk) and found that the fat content of milk did not play a role. After the addition of whole milk that has the largest amount of fat, the anti-mutagenic effect of tea was reduced the least compared to the semi-skimmed and skimmed milk [15]. In this study, where a non-fat dairy product was used for the 3.5 MP conditions, the effect of the green tea that was present with water but without 3.5 MP was completely inhibited. 

The quantity of milk protein for the 15 MP conditions is sufficient to increase DIT itself, yet also inhibits an additional effect of the green tea. The quantity of milk protein was 15 energy% of the breakfast. On average this was 16.1 g for each subject, which usually is considered to be a normal protein intake. The explanation that casein forms complexes with the polyphenols and thereby slows the emptying of the stomach because casein coagulates under the acidic condition of the stomach, is the most acceptable one [30]. Finally, it might be possible that protein-polyphenol complexes form metabolites that are taken up in the small intestine similar to the GT metabolites but do not have the same effect. This, however, has never been studied and should be investigated. 

Limitations of the study were the substantial dropout of 17 subjects after the 15 MP conditions and, although randomization took place within, subjects first received the 15 MP conditions and thereafter the 3.5 MP conditions.

## 5. Conclusions

In summary, in line with the results from our previous study where green tea in addition to a high-protein diet had no additive effect on weight maintenance over the long-term, supplementation of a combination of green tea and milk protein in different amounts did not have an additional effect on diet-induced thermogenesis. This can be explained by the inhibitory effects of milk protein on the effects of green tea on DIT. 
